# A novel missense heterozygous mutation in *MAP3K1* gene causes 46, XY disorder of sex development: case report and literature review

**DOI:** 10.1002/mgg3.1514

**Published:** 2020-09-28

**Authors:** Aisha Al Shamsi, Noura Al Hassani, Moustafa Hamchou, Raya Almazrouei, Aziz Mhanni

**Affiliations:** ^1^ Genetic Division Pediatrics Department Tawam Hospital Al Ain United Arab Emirates; ^2^ Endocrine Division Pediatrics Department Tawam Hospital Al Ain United Arab Emirates; ^3^ Pediatric surgery Division Surgery Department Tawam Hospital Al Ain United Arab Emirates; ^4^ Endocrine Division Internal Medicine Department Tawam Hospital Al Ain United Arab Emirates; ^5^ Department of Pediatrics & Child Health Max Rady College of Medicine Rady Faculty of Health Sciences University of Manitoba Winnipeg Canada

## Abstract

**Background:**

Disorders of sex development (DSD) can result from congenital defect in sex determining pathway. Mitogen‐activated protein kinase kinase kinase 1 (*MAP3K1*) is one of the commonest genes that has been identified to cause 46, XY DSD. It can present as complete or partial gonadal dysgenesis even within the same kindred. Few mutations in this gene have previously been identified in a high proportion of individuals with 46, XY gonadal dysgenesis.

**Methods and Results:**

We report three siblings with same novel variant in *MAP3K1* gene presenting with variable degrees of partial gonadal dysgenesis. Clinical and genetic assessments were performed for the three siblings, while endocrine evaluation was done for two of them. The identified mutation (p.Thr657Arg) was previously classified as a pathogenic variant, although apparently there are no reported humans with this mutation.

**Conclusion:**

This report adds to the genotype‐phenotype correlation, highlighting the clinical importance of considering *MAP3K1* gene defects as part of the differential diagnosis for complete or partial gonadal dysgenesis especially with multiple affected family members.

## Abstract 2

We describe in detail the clinical phenotypes of three affected siblings, with same novel variant in *MAP3K1* gene presenting with variable degrees of partial gonadal dysgenesis. In this report, we declare the identification of a new disease causing missense variant in *MAP3K1* gene which was not apparently described in humans before. Our report adds to the genotype‐phenotype correlation, highlighting the clinical importance of considering *MAP3K1* gene defects as part of the differential diagnosis for complete or partial gonadal dysgenesis especially with multiple affected family members.

## 1 INTRODUCTION

Disorders of sex development (DSD) are congenital conditions in which development of chromosomal, gonadal, or anatomic sex is atypical (Granados et al., 2017). There are no clear estimates of the incidence rate of subjects presenting with ambiguous genitalia at birth, however, it has been estimated to be approximately 1 in 4500–5500 birth. The incidence rate among subjects with 46, XY to have a DSD has been estimated to be 1 in 20,000 births (Lee et al., 2016). The process of sex development in human embryo composed of three stages. The indifferent stage, from fertilization to 6 weeks gestation, is where both XY and XX embryos are morphologically indistinguishable. This is followed by sex determination stage where the bipotential gonads form testes or ovaries. This stage is controlled by several determining genes. These genes comprise two signaling pathways, *SRY* (NM_003140.2) and its downstream target *SOX9* (NM_000346.3) in the testis determination and *WNT4*‐*CTNNB1* (β‐catenin, NM_001098209.1) signaling pathway in the ovary determination (León et al., 2019; Xue et al., 2019). The expression of the *SRY* gene on the Y chromosome causes the undifferentiated gonad to develop into a testis. Whereas in the absence of a Y chromosome and expression of *SRY*, the undifferentiated gonad develops as an ovary and the Wolffian ducts regress (Ono & Harley, 2013). The third stage is sex differentiation where the gonads produce hormones that differentiate the internal and external genitalia (León et al., 2019). Sertoli cells in the developing testis produce Anti‐Müllerian Hormone (AMH), leading to regression of the Müllerian ducts, while production of testosterone by the Leydig cells promotes differentiation of Wolffian duct structures into male internal genitalia.

DSDs are classified into three subclasses: sex chromosome DSDs, 46, XY DSDs and 46, XX DSDs. The 46, XY DSDs comprise disorders of gonadal development, disorders of androgen synthesis and action, persistent Müllerian duct syndrome, and other unclassified disorders (León et al., 2019). Most individuals with DSD lack a known specific genetic aetiology; however, exome sequencing has been used to identify a likely genetic aetiology in up to 43% of patients with 46, XY DSD (Baxter et al., 2015; Eggers et al., 2016). Here, we report three siblings with a heterozygous missense *MAP3K1* variant causing variable degrees of 46, XY partial gonadal dysgenesis.

## 2 PATIENTS AND METHODS

### 2.1 Ethical compliance

This study was approved by our regional research ethical committee. Ref. No.: AA/AJ/733.

### 2.2 Patients

**FIGURE 1:**
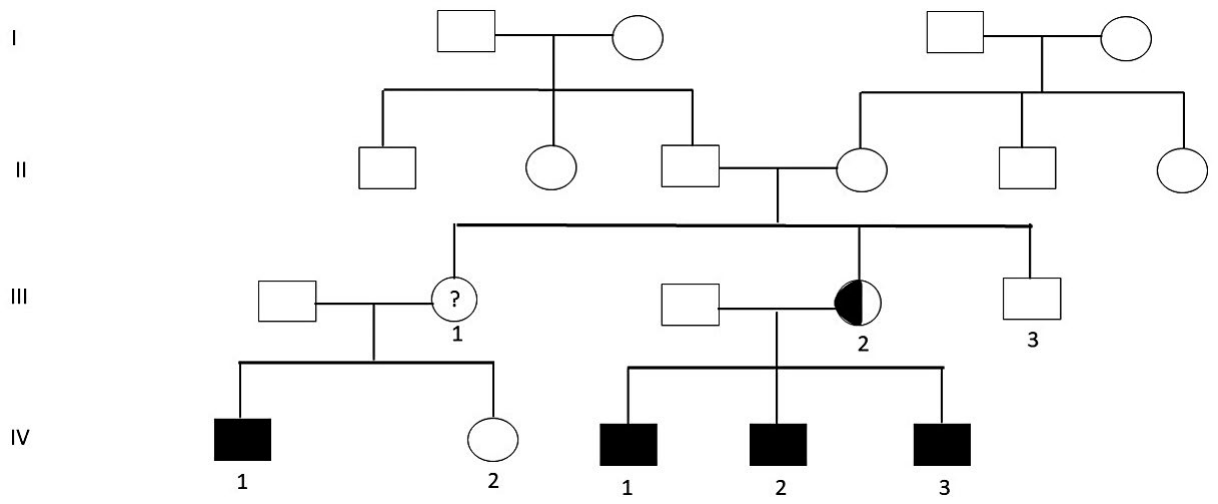
Family pedigree. Circles and squares are females and males, respectively; filled symbols are affected members; half‐filled symbols are carrier members; Roman numbers indicate the generations; and Arabic numbers indicate offspring

Three siblings with ambiguous genitalia, were evaluated by the Genetic and Endocrine teams at Tawam Hospital, Al Ain, United Arab Emirates. Molecular characterization of the aetiology was made possible after one of the siblings referred for genetic evaluation before proceeding with surgical correction. Those siblings were born to a non‐consanguineous couple (Figure 1). There was no history of miscarriages, neonatal deaths, infertility, developmental delay, or endocrine or sex reversal disorders. A maternal first cousin was reported to have had hypospadias, but no other information was available about him.

### 2.3 Endocrine evaluation

All hormonal investigations done using chemoluminescence immunoassay tested on Roche 8000i. Normal reference range of the hormonal levels in this paper was as reported for age by our laboratory.

### 2.4 Whole exome sequencing

Blood samples were collected from the patients and the parents after written informed consent was obtained. DNA was extracted from peripheral white blood cells using Flexigene DNA extraction kit (Qiagen Gmbh, Germany) according to the manufacturer's protocol. Whole‐exome sequencing was carried out by CENTOGENE AG laboratory in Rostock, Germany (www.cento​gene.com). The sample was processed on the Ion Proton Platform (Life Technologies, Renfrew, United Kingdom). Approximately 36.5 Mb of coding exons were converted as described by Consensus Coding Sequences. An in‐house bioinformatics pipeline, including read alignment to GRCh37/hg19 genome assembly, variant calling (single nucleotide and small deletion/insertion variants), annotation and comprehensive variant filtering is applied. All variants with minor allele frequency (MAF) of less than 1% in gnomAD database, and disease‐causing variants reported in HGMD^®^, in ClinVar or in CentoMD^®^ are considered. The investigation for relevant variants is focused on coding exons and flanking ±20 intronic nucleotides of genes with a clear genotype phenotype correlation (based on OMIM^®^ information).

## 3 RESULTS

### 3.1 Clinical description

**FIGURE 2:**
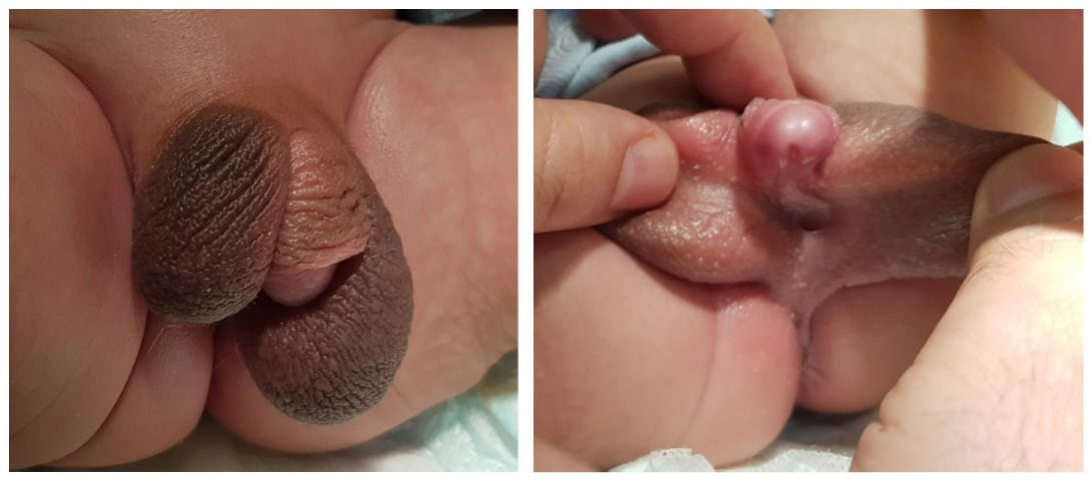
Patient's 1 genitalia, consisting of perineal hypospadias, severe chordee and bifid scrotum with both testes palpable in the scrotum

*Patient 1* is a 2‐year‐old and 6‐month‐old male, born at term by a Caesarean section due to previous Caesarean section, with a birth weight of 3160 g (28th%ile), a length of 51 cm (40th%ile) and a head circumference of 36 cm (45th%ile). His antenatal history was unremarkable. At birth he was noted to have perineal hypospadias with penile length of 3 cm with severe chordee and bifid scrotum with both testes palpable in the scrotum (Figure 2). His scrotum ultrasound visualized testicles within the scrotal sac and described as more of retractile testicles. His pelvic and abdominal ultrasound were reported as normal with no abnormal internal structures. His initial electrolytes were normal with acceptable baseline gonadotropin and androgen levels for his age (4 days old): Follicle‐stimulating hormone (FSH) 7.3 mIU/ml (normal for age ≤3.3), Luteinizing hormone (LH) 6.0 mIU/ml (normal for age ≤0.4), testosterone (T) 2.78 nmol/L (normal for age 13.6–26.0), dihydrotestosterone (DHT) 126 pg/ml (normal for age ≤1200), dehydroepiandrosterone sulfate (DHEAS) 9.7 µmol/L (normal for age 2.93–16.5), and androstenedione 11 nmol/L (normal for age 1–11.5). Adrenocorticotropic hormone (ACTH) 19 pmol/L (normal range 0–10.1), and 17 hydroxyprogesterone 15.4 pmol/L (normal range 1–10) which were mildly high and normalized by 1 month of age while Anti‐Müllerian Hormone (AMH) was 116.1 pmol/L (reference range 5.5–103). As T level was not matching with higher level of LH and FSH, Beta human chorionic gonadotropin (HCG) stimulation test was done at 3 weeks of age using 500 IU daily for 3 days and it showed a significant increase in both T level and DHT level up to 13.08 nmol/L and 1140 pg/ml, respectively, with T/DHT ratio of <12. Chromosomal karyotype confirmed 46, XY and chromosomal microarray was normal. Whole exome sequencing showed a variant NM_005921.2 (MAP3K1):c.1970C>G (p.Thr657Arg) in exon 11 of *MAP3K1* gene causing an amino acid change from Threonine to Arginine at position 657. This variant was classified as a variant of uncertain significance according to ACMG guidelines, which was reclassified recently as pathogenic variant by Chamberlin et al. (2019). He underwent surgical correction for his ambiguous genitalia as two stages repair at 14 months and 20 months of age.

*Patient 2* is a 4‐year‐old male, born at term by Caesarean section due to failed induction of labor, with a birth weight of 3270 g (20th%ile), a birth length of 53 cm (79.9th%ile) and a head circumference of 36 cm (45th%ile). The pregnancy history was remarkable for controlled gestational diabetes on diet and an antenatal ultrasound showed mild to moderate polyhydramnios. After delivery, he was found to have coronal hypospadias (with normal penial length; 3.5 cm) with severe chordee and both testes palpable within normally developed scrotum. He was nondysmorphic. The patient was not assessed at that time by either one of our teams; Endocrine or Genetics, that's why no hormonal studies and anatomical imaging of the internal anatomy were done that time. He was assessed by Pediatric Surgery and undergone hypospadias and chordee repair surgery at age of 16 months. At 2 years of age, he had another surgery for incomplete circumcision and retention penile cyst. He was evaluated in the Genetics clinic after his younger sibling (patient 1) evaluation and he was found to have the same variant in the *MAP3K1* gene.

**FIGURE 3:**
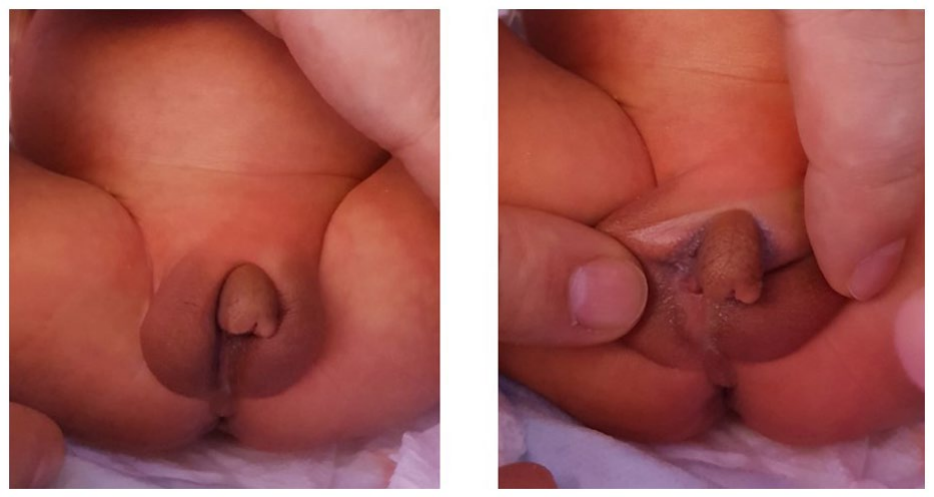
Patient's 3 external genitalia, consisting of micropenis, perineal urethra, no vaginal opening with bifid empty scrotum, and no palpable gonads

**FIGURE 4:**
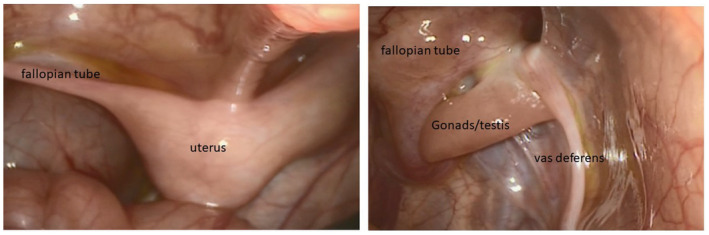
Intra‐operation findings of patient 3

*Patient 3*, the youngest sibling in this family, is 7 months old. He was born at term by an emergency Caesarian section due to fetal distress with a birth weight of 2800 g (8.38th%ile), a birth length of 46.5 cm (5th%ile) and a head circumference of 35 cm (26.4th%ile). After birth, he was found to have micropenis (length of 1.5 cm), perineal urethra, no vaginal opening with bifid empty scrotum, and no palpable gonads (Figure 3). A pelvic ultrasound showed a uterus/cervix like structure seen posterior to the urinary bladder measuring grossly about 2.4 × 1.4 × 1 cm with no ovaries visualized. The external genitalia were scanned showing soft tissue echogenicity structures surrounded by minimal fluid, most likely represent bilateral inguinal hernias more on the right side, likely containing bowel. Midline soft tissue structure, most likely represent enlarged clitoris. No definite penile or testicular structures could be seen by the ultrasound. His initial electrolytes were normal. At 1 week of age, baseline AMH was 93.3 pmol/L, LH was 0.8 mIU/ml and FSH was 3.6 mIU/ml. In link with that, estradiol was 47 pmol/L (normal range 41.4–159), random testosterone 1.99 nmol/L (normal range 13.6–26). Adrenal workup revealed no abnormalities that could highlight a steroid synthesis defect: 17‐hydroxyprogesterone 35.4 nmol/L, ACTH 13.4 pmol/L, DHEA 3.18 µmol/L, and androstenedione 9.13 nmol/L. At 2 weeks of age, beta HCG stimulation test with 500 units daily for 3 days showed suboptimal response with T level change from baseline of 1.04 nmol/L to post‐stimulation level of 2.5 nmol/L, while the DHT was undetectable <50 pg/ml at baseline and increased post‐stimulation to 149 pg/ml. Diagnostic laparoscopy and bilateral inguinal hernia repair showed that the right gonad was inside the hernial sac and the left gonad was close to the internal ring. The patient has what looks like normal uterus with two fallopian tubes which were close to the gonads (Figure 4). The two gonads were mobilized inside the abdomen by pulling the gonads outside the hernial sac, adhesions were released and hernia was repaired. No biopsy was taken from the gonads.

Chromosomal karyotype confirmed 46, XY. Fluorescence in Situ Hybridization (FISH) was applied using DXZ1 and DYZ3, DNA probes, specific for chromosomes X and Y, respectively; it showed one signal was obtained from each of chromosomes X and Y specific DNA probes, indicating a male sex compliment. Molecular testing revealed that he also has the same variant, c.1970C>G p.(Thr657Arg) in *MAP3K1* gene.

Parental testing confirmed that the mother has the c.1970C>G p.(Thr657Arg) in *MAP3K1* gene variant while the father is negative. The aunt who has a child with reported hypospadias has not yet been tested, neither has her son.

## 4 DISCUSSION

Gonadal dysgenesis is one of 46, XY DSD causes. It is characterized by incomplete or defective formation of the gonads (ovary or testis) due to either structural or numerical anomalies of the sex chromosomes or mutations in the genes involved in the development of the gonads (McCann‐Crosby et al., 2014). According to the gonadal morphology, it can be classified as either complete gonadal dysgenesis (CGD) or partial gonadal dysgenesis (PGD). Although 45,X/46, XY is the most common karyotype seen in PGD, 46, XY and other form of mosaicism involving the Y chromosomes can also be seen (McCann‐Crosby et al., 2014).

Along with the *SRY* gene, several genes and signaling pathways involved in sex determination associated with a wide phenotypic spectrum of DSD have been identified. Mitogen‐activated protein kinase kinase kinase 1 (*MAP3K1*; NM_005921.2) and MAPK‐signaling pathway is one of the genetic pathways that control normal development of the testes. Recent studies suggest that *MAP3K1* is one of the more commonly mutated genes in 46, XY DSD, with mutations reported in 13%–18% of patients (Ostrer, 2014). The gene consists of 20 exons in length and the reported mutations occurred at well‐conserved sites in exons 2, 3, 13, and 14 and have the characteristic of being in‐frame alterations, either non‐conservative single‐nucleotide variants or familial splice acceptor site variants that resulted in in‐frame insertions or deletions. (See Table 1). The net result of these mutations is gain‐of‐function effect, causing increased phosphorylation of the downstream targets, p38 and ERK1/2, and increased binding of cofactors *RHOA* (NM_001313941.1), *MAP3K4* (NM_001291958.1), *FRAT1* (NM_005479.3), and *AXIN1*. These downstream effects tilt the balance of gene expression in the testis‐determining pathway causing decreased expression of *SOX9* and its downstream targets, *FGF9* (NM_002010.2) and *FGFR2* (NM_022976.1), and increased expression of B‐catenin and its downstream target, *FOXL2* (NM_023067.3) (Ostrer, 2014). Adding new insights into MAPK related DSD pathogenesis, it has been reported that effects on protein binding depend on the domain in which the mutations occur (Chamberlin et al., 2019).

**TABLE 1:** Reported mutations in *MAP3K1* gene

DNA change	Protein change	Number of patients	Reference
c.634‐8T>A	—	10	Pearlman et al. (2010)
c.1846G>A	p.Gly616Arg	5	
c.566T>G	p.Leu189Pro	2	
c.2416G>A	p.D806N	4	Das et al. (2013)
c.3084A>G	p.Q1028Q	1	
c. 1284G>A	p.T428T	1	
c. 2822_2824insCAA	p. 942insT	2	
c.458C>T	p.Pro153Leu	1	Loke et al. (2014)
c.2180‐2A>G	p.Gly727_Ile761del	1	
c.1846G>A	p.(Gly616Arg)	2	Baxter et al. (2015)
c.1016G>A	p.(Arg339Gln)	1	
c.770C>T	p.(Pro257Leu)	1	
c.566T>G	p.(Leu189Arg)	2	Eggers et al. (2016)
c.2071T>C	p.(Cys691Arg)	1	
c.4328C>T	p.(Ala1443Val)	2	
c.934A>T	p.(Met312Leu)	4	
c.710A>G	p.(Gln237Arg)	1	
c.394G>C	p.(Asp132His)	1	
c.14_16insCGG	p.(Ala5dup)	2	Granados et al. (2017)
c.1760T>A	p.(Leu587His)	2	
c.566T>A	p.(Leu189Gln)	1	
c.2291T>G	p.(Leu764Arg)	1	
c.2072G>A	p.(Cys691Tyr)	2	Kopylova IV et al. (2018)
c.2858_2872del CAACAACAACAACAA	p.944_948del	1	
c.2117T>G	p.(L706R)	1	Xue et al. (2019)
c.1970C>G	p.(Thr657Arg)	3	*Current report* Chamberlin et al. (2019)^a^

a Our mutation is tested to be pathogenic as per this study.

*MAP3K1* mutations were first described in 2010 in two large families with 46, XY DSD in an autosomal dominant, sex‐limited pattern of transmission, as well as in 2 of 11 sporadic cases of 46, XY gonadal dysgenesis. In the large families that were reported, there were variable phenotypic presentations ranging from complete gonadal dysgenesis, sometimes with gonadoblastoma, to partial gonadal dysgenesis like hypospadias and micropenis with cryptorchidism (Pearlman et al., 2010). 46, XX individuals carrying pathogenic variants in *MAP3K1* gene are completely asymptomatic. Therefore, it was recommended by Granados and his colleagues that testing for *MAP3K1* variants should be considered in patients with 46, XY complete or partial gonadal dysgenesis, particularly in families with multiple affected individuals with 46, XY DSD (Granados et al., 2017).

Table 1 summarizes all reported mutations of *MAP3K1* gene and the number of cases with each one. The variant (c.634‐8T>A) is so far the most common variant in *MAP3K1* associated DSD. We are reporting a heterozygous missense *MAP3K1* variants in three siblings causing partial gonadal dysgenesis with variable severities. To the best of our knowledge, this variant is reported for the first time in human and both family segregation analysis and endocrine hormonal functional studies are strongly suggestive that this variant is pathogenic. Recently, this variant was reported to be pathogenic by Chamberlin et al. (2019) who used actual patient samples, structural modeling, and functional data to understand alterations of the *MAP3K1* protein and the effects on protein folding, binding and downstream target phosphorylation.

Managing these patients will require a multidisciplinary team approach to address the urgent medical, surgical, and psychological needs. Sex assignment is a critical management issue that needs addressing. Although this might be obvious in some patients, such as in Patient 2, it might be a more challenging decision as in patient 3 with female internal organs and poor Beta HCG stimulation test response which later on may necessitate revising the male sex rearing chosen by the family.

## 5 CONCLUSION

Our three reported siblings have a novel heterozygous missense variant in *MAP3K1* gene expanding the spectrum of pathogenic mutations causing 46, XY gonadal dysgenesis. Furthermore, our report adds to the genotype‐phenotype correlation, highlighting the clinical importance of considering *MAP3K1* gene defects as part of the differential diagnosis for complete or partial gonadal dysgenesis especially with multiple affected family members.

## CONFLICT OF INTERESTS

The authors declare that they have no competing interests.

## AUTHORS’ CONTRIBUTION

AA‐S, NA‐H, MH, RA, and AM contributed to conception and design. AA‐S drafted the manuscript. All authors contributed to acquisition, revised manuscript, and agreed to be accountable for all aspects of work ensuring integrity and accuracy. All authors read and approved the final manuscript.

## CONSENT FOR PUBLICATION

Consent for publication was obtained from the parent of the reported patients.
